# Polarization-Resolved Position-Sensitive Self-Powered Binary Photodetection in Multilayer Janus CrSBr

**DOI:** 10.1021/acsami.3c13552

**Published:** 2023-12-26

**Authors:** Jaganandha Panda, Satyam Sahu, Golam Haider, Mukesh Kumar Thakur, Kseniia Mosina, Matěj Velický, Jana Vejpravova, Zdeněk Sofer, Martin Kalbáč

**Affiliations:** † J. Heyrovský Institute of Physical Chemistry, Dolejskova 3, 182 23 Prague 8, Czech Republic; ‡ Department of Biophysics, Chemical and Macromolecular Physics, Faculty of Mathematics and Physics, Charles University, Ke Karlovu 3, 121 16 Prague 2, Czech Republic; § Department of Inorganic Chemistry, University of Chemistry and Technology Prague, Technicka 5, 166 28 Prague 6, Czech Republic; ∥ Department of Condensed Matter Physics, Faculty of Mathematics and Physics, Charles University, Ke Karlovu 5, 121 16 Prague 2, Czech Republic

**Keywords:** optical polarization detection, Janus 2D layer, CrSBr, self-powered photodetector, position sensitivity

## Abstract

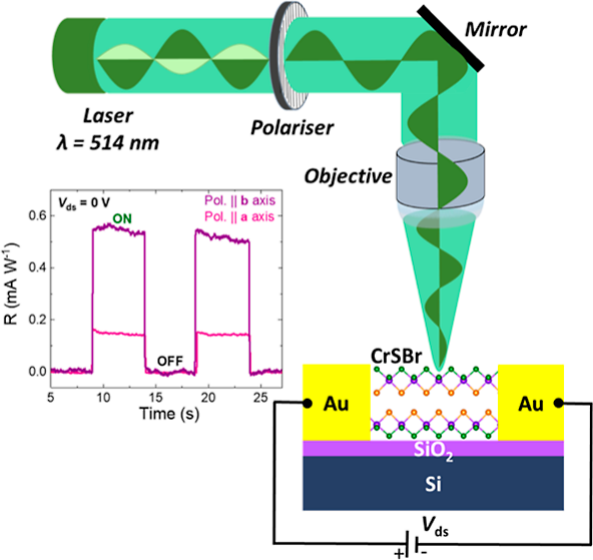

Recent progress in polarization-resolved photodetection based on low-symmetry 2D materials has formed the basis of cutting-edge optoelectronic devices, including quantum optical communication, 3D image processing, and sensing applications. Here, we report an optical polarization-resolving photodetector (PD) fabricated from multilayer semiconducting CrSBr single crystals with high structural anisotropy. We have demonstrated self-powered photodetection due to the formation of Schottky junctions at the Au–CrSBr interfaces, which also caused the photocurrent to display a position-sensitive and binary nature. The self-biased CrSBr PD showed a photoresponsivity of ∼0.26 mA/W with a detectivity of 3.4 × 10^8^ Jones at 514 nm excitation of fluency (0.42 mW/cm^2^) under ambient conditions. The optical polarization-induced photoresponse exhibits a large dichroic ratio of 3.4, while the polarization is set along the **a-** and the **b**-axes of single-crystalline CrSBr. The PD also showed excellent stability, retaining >95% of the initial photoresponsivity in ambient conditions for more than five months without encapsulation. Thus, we demonstrate CrSBr as a fascinating material for ultralow-powered optical polarization-resolving optoelectronic devices for cutting-edge technology.

## 1 Introduction

Photodetectors (PDs) are ubiquitous electronic devices in modern technology that harvest optical signals and convert them into electrical responses.^1−5^ Depending on the device architecture, PDs usually require an external bias to assist the directional movement of the photogenerated charge carriers (electrons and/or holes) to generate photocurrent.^6,7^ However, by engineering the energy bands of the constituent materials, an internal electric field at their interface can be produced and utilized as the driver for the photo-generated carriers. Self-powered PDs operate under zero externally applied bias and are deemed technologically important for low-power electronics such as image sensing and optical communications.^8,9^ Thus, self-powered PDs not only reduce the cost of the devices but also greatly reduce the size of the whole integrated system.^10−14^ The traditional approach to designing self-powered PDs is the p–n homojunction assembly, heterojunctions, and Schottky junctions by taking advantage of their photovoltaic effects.^12,15−18^ Among these junctions, metal/semiconductor interfaces have prompted growing scientific interest. However, the construction of Schottky junction PDs is limited by the type of 2 dimensional (2D) material and metal electrodes, and it is necessary to find a pair of suitable metal electrodes for asymmetrical contact engineering.^17,19−22^

On the other hand, polarization-sensitive PDs are demanding in state-of-the-art technology including quantum computing, efficient three-dimensional object detection in light detection and ranging (LiDAR) devices, high-density optical signal processing, navigation, and high-contrast polarizers.^8,9^ Detection of optical polarization utilizing optically anisotropic materials has attracted increasing research interest in recent years. Low-charge symmetry crystals such as layered van der Waals materials including black phosphorus, GaTe, GeS, SnS, SnSe, ReSe_2_, ReS_2_, and GeAs_2_, exhibit in-plane optical anisotropy and birefringence making them promising materials for the detection of optical polarization.^23−29^ However, poor air stability and oriented growth are among the several shortcomings that limit their performance and practicality.^2,30^ The investigation of stable low-symmetry 2D materials and their polarization-selective light–matter interaction and the interplay between their layer thickness, structural, and optical anisotropy are still in their early stage.^31^ Recently discovered Janus materials provide additional freedom to introduce an optically anisotropic response in monolayer 2D materials. In particular, newly prepared CrSBr exhibits an air-stable optically anisotropic semiconducting nature with a band gap of 1.5 eV.^32−34^ The exfoliated CrSBr yields elongated flakes along the **b**-axis with a high aspect ratio. This peculiarity in the exfoliated flakes causes in-plane optical anisotropy and directional dependence that can affect the electron–phonon and light–matter interactions.^32−34^ Moreover, the puckered crystal structure of CrSBr is a crucial feature, causing the breaking of the sublattice symmetry for optical anisotropy and thus resulting in layer-dependent polarization-sensitive properties.^23,32,35,36^

In this work, we address several aforementioned challenges in a rather simply designed device based on a few layers of CrSBr in a linear Au/CrSBr/Au architecture. While CrSBr enables a strongly polarization-sensitive photoresponse, the Schottky potential developed in both Au/CrSBr and CrSBr/Au facilitates a self-power device that exhibits a position-sensitive binary photoresponse. We have systematically investigated the polarization-resolved and self-powered device performance under a series of excitation sources from 488 to 633 nm demonstrating broadband photoresponse. The position sensitivity of the photocurrent has been found to be ∼0.37 nA/μm under 514 nm excitation of intensity 1.41 mW/cm^2^. Additionally, a highly polarization-sensitive photoresponse originating from the polarization-sensitive light–matter interaction has been studied. The optical polarization-dependent photocurrent was further consolidated by the statistical average over several samples, evaluating device performance of CrSBr on different substrates and over a long time (150 days) as well as employing spectroscopic characterization such as Raman and photoluminescence (PL) spectroscopy. Our work thus provides a viable route to fabricate high-performance self-biased polarization-sensitive PDs, avoiding the complex requirement of device fabrication and interface engineering.

## 2 Methods

### 2.1 Device Fabrication

The single-crystal CrSBr PD was fabricated by a simple, dry transfer method, with a highly p-doped silicon wafer covered with 300 nm thick SiO_2_ used as a substrate. The metal electrode patterns were fabricated on the SiO_2_/Si substrate by direct lithography (MicroWriter, Durham Magneto Optics Ltd.) followed by metal lift-off. For the electrode, optimized layers of Cr (5 nm) and Au (45 nm) metals were deposited on the silicon substrate by sputtering. Finally, lift-off was performed in hot acetone and rinsed by IPA. Next, a multilayer CrSBr was exfoliated on a thin PDMS membrane from the bulk counterparts using Nitto tape. The targeted CrSBr flake on the PDMS was transferred onto the patterned silicon substrate using the micropositioning arm of a transfer stage. The device was imaged using an optical microscope, with the optical parameters adjusted to observe the contrast of CrSBr with SiO_2_/Si.

### 2.2 Device Characterization

Raman spectra were recorded at room temperature by a WITec micro-Raman system (alpha300R) equipped with a 532 nm laser. The thickness of the exfoliated layers was obtained by AFM (Bruker, Dimension Icon). The electrical properties of the fabricated devices were measured in a homemade probe station (HFS600E-PB4) integrated with a source meter (Keithley 2610). To perform photocurrent measurements, the 514 nm laser was focused on the device with a 100× objective, and the laser spot was nearly flat with a size of about ∼500 nm. For polarization-dependent measurements, we used a half-wave plate, which can turn an incident unpolarized light into polarized light, and was placed between the light source and the PD. The power and polarization of the incident light were controlled by using a variable neutral density filter and the half-wave plate, respectively. We first checked the polarization direction of the laser with an optical polarimeter (Thorlabs), which showed a perfectly polarized laser signal, which we referenced with the crystalline axes of the samples utilizing the optical microscope and a manual goniometer. The data for different polarization angles were obtained by changing the goniometer angle.

All of the measurements were carried out at room temperature at an ambient environment.

## 3 Results and Discussion

### 3.1 Materials and Device Design

**Figure 1:**
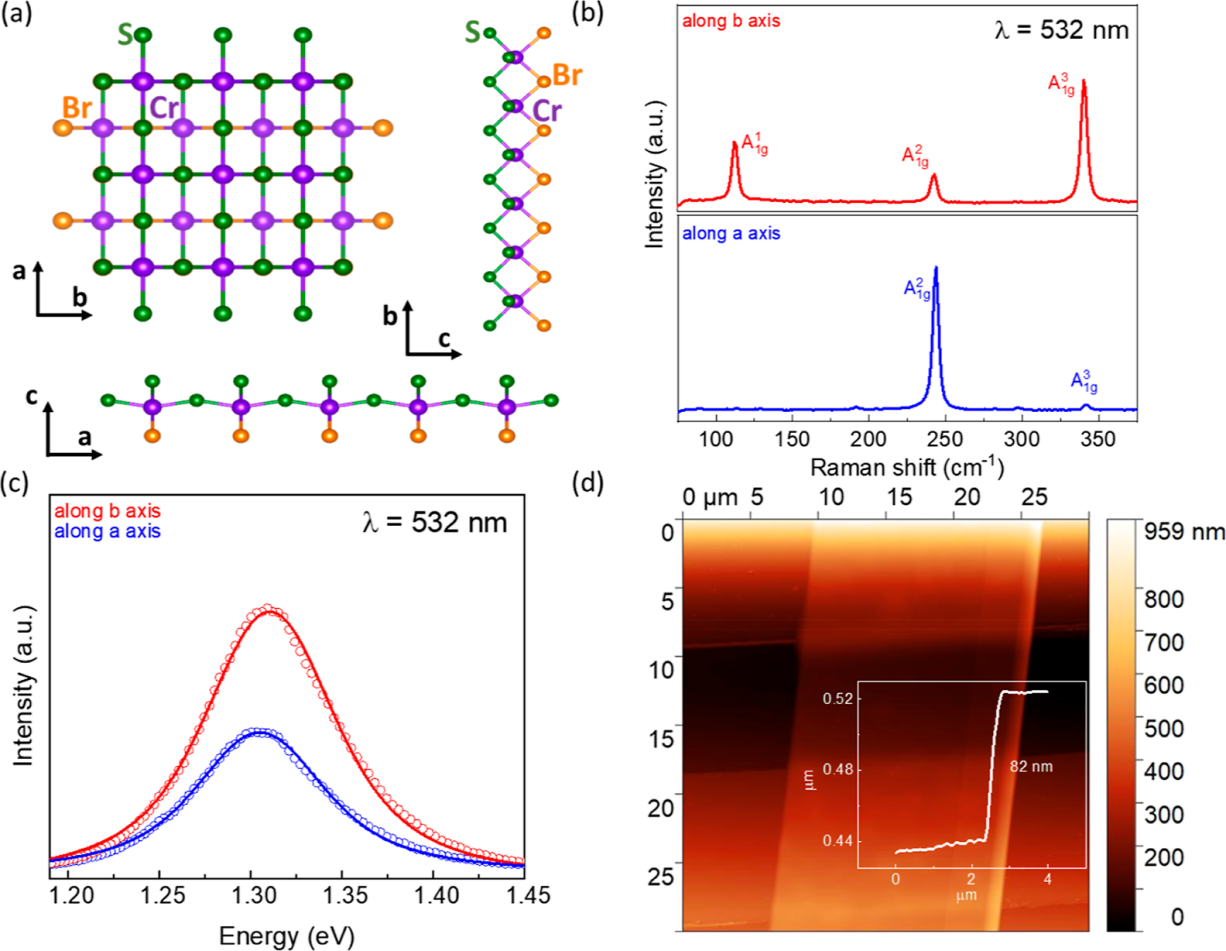
Basic characterizations of CrSBr. (a) Crystal structure of CrSBr. The left image shows a top view from the **c**-axis, the right image shows a side view from the **b**-axis, and the bottom image shows a side view along the **a**-axis. Purple, green, and orange represent the Cr, S, and Br atoms, respectively. (b) Raman spectra of the exfoliated few-layer CrSBr on SiO_2_/Si substrate measured at ambient conditions with the excitation laser (532 nm) polarized along the **a** and **b** axis. (c) PL spectra of the same flake (empty circles correspond to raw data and solid lines are the Voigt fitting data). (d) AFM image of the exfoliated CrSBr flake on the top of the Au electrodes and corresponding height profile with a thickness of 82 nm.

Multilayer CrSBr flakes were mechanically exfoliated from a bulk crystal grown by the chemical vapor transport technique in quartz ampoule from elements.^37^Figure 1a shows the layered crystal structure of CrSBr, which belongs to the *Pmmn* (*D*_2h_) orthorhombic space group. Each buckled CrS plane is sandwiched between the top and bottom Br atoms, and these layers stack through the van der Waals interaction along the **c**-axis. To explore the nature of the phonon modes, Raman spectroscopic characterization of the crystal was performed by using a 532 nm excitation laser wavelength. Figure 1b shows the typical Raman spectra of an ∼82 nm CrSBr flake, with the incident laser linearly polarized along the **a** and **b** crystalline axes. Three primary Raman peaks, assigned as P_1_ (≈110 cm^–1^) for the A_1g_^1^ phonon mode, P_2_ (≈245 cm^–1^) for the A_1g_^2^ mode, and P_3_ (≈343 cm^–1^) for the A_1g_^3^ mode, were observed along the **b**-axis (as discussed later). Only a single peak at 245 cm^–1^ was found in the spectra measured with the polarization along the **a**-axis, consistent with previous reports.^38^ All Raman peaks correspond to the out-of-plane A_1g_ vibrational modes, which aligns with the literature.^38^ The PL spectra under 532 nm excitation at room temperature show a strong emission centered at around 1.31 eV (Figure 1c), which originates from the band-edge emission rather than localized ligand-field luminescence.^38−40^ The thickness-dependent Raman and PL spectra of the CrSBr are shown in Figure S1 of the Supporting Information, corroborating previous reports.^34,38^ The exact thickness of the flakes was determined by AFM topography. Figure 1d shows a typical AFM image of exfoliated few-layer CrSBr transferred on prepatterned gold electrodes on the SiO_2_/Si substrate, which was further used as a phototransistor device at the ambient conditions.

### 3.2 Optoelectronic Characterization of the Device under Unpolarized Photon Flux

**Figure 2:**
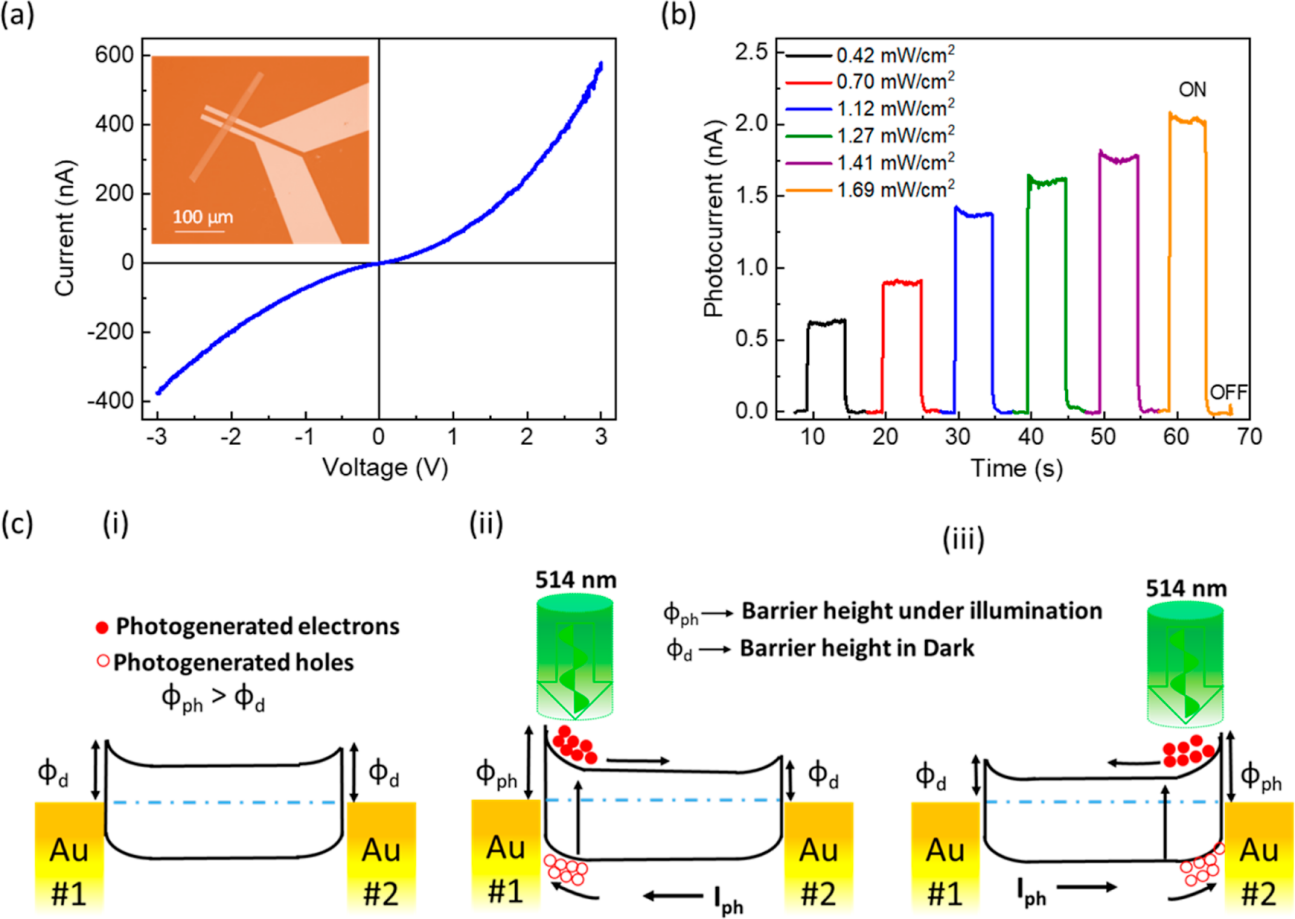
Self-biased optoelectronic properties of an 82 nm CrSBr flake. (a) *I*–*V* characteristics of the device under dark conditions. The inset shows the optical image of the device. (b) Transient photoresponse of the device under the excitation of different power densities at the 0 V applied bias. (c) Schematic illustration of the energy band diagram of the Au–CrSBr–Au Schottky junction device, where different electrodes have been marked. The mismatch between the CrSBr and Au work functions under thermal equilibrium generates a built-in electric field at the Au–CrSBr and CrSBr–Au interfaces of similar values but along opposite directions in the dark. Inhomogeneous photon illumination to the junctions however causes a transient change of local work function at the illuminated area of CrSBr due to the photogenerated electrons, which results in variation of the magnitudes of the transient built-in electric field that drives the photocurrent. Panel (i) shows the energy band diagram under thermal equilibrium in the dark, and (ii) and (iii) show the transient variation of the electric field under the illumination of photon flux to the electrode Au#1–CrSBr and CrSBr–Au#2 interfaces, respectively.

The current–voltage (*I–V*) characteristics of the device under dark conditions are shown in Figure 2a; the inset shows an optical microscopy image of the device. The *I–V* curve confirms a typical nonlinear diode-like characteristic, indicating the formation of Schottky junctions at the interface of semiconducting CrSBr and Au electrodes.^41,42^ The *I*–*V* characteristics of CrSBr under dark and light of wavelength 514 nm have been shown in Supporting Information Figure S2. To elucidate the self-powered photodetection properties of the device, the transient device current was recorded under periodic illumination of a 514 nm laser at different excitation intensities without applying any external bias (*V*_SD_ = 0 V). Figure 2b shows the transient net photocurrent (*I*_ph_) of the device measured under varying excitation power, where the net photocurrent is defined as the difference between the net device current under illumination (*I*_l_) and dark conditions (*I*_d_), that is, *I*_ph_ = *I*_l_ – *I*_d_. It can be seen that the photocurrent increased with the illumination power, which is consistent with the previous measurements of both self-powered devices and devices to which an external bias was applied.^43−45^ The photocurrent is generated purely due to the contribution of the potentials generated at the electrode/CrSBr interface. Importantly, the generated potential has opposite polarities, further limiting the circuit’s photocurrent. Thus, the obtained photocurrent is not comparable with traditional *p–n* junction-based self-biased devices. However, in order to obtain a qualitative comparison of the device performance figure of merit of this device, we performed an external bias-dependent photocurrent measurement as shown in Figure S3 in the Supporting Information, which shows a photocurrent value up to 20 nA under the excitation of 1.4 mW/cm^2^ power density. The self-powered behavior of the device originates from the lateral photovoltaic effect due to a built-in electric field resulting from the band offset at the metal–semiconductor Schottky junction (Au–CrSBr and CrSBr–Au) interfaces.^44,46,47^ Note that both of the electrodes forming the Schottky junction have opposite built-in electric fields (Figure 2c (i)), which should nullify the photocurrent for uniform illumination over the whole device area. However, in our confocal measurement setup, the excitation laser spot size (of diameter ∼500 nm) is smaller than the device active area (length 5 μm), which enabled us to map the photocurrent over the device area by selectively exciting the specific Schottky junction associated with the individual electrodes (Figure 2c (ii) and (iii)). In this situation, the photoexcited electrons cause a transient change of band offset at the illuminated Schottky junction, which drives the photogenerated carriers contributing to the photocurrent. Here, the second Schottky junction remains as a passive resistor to the photocurrent, which partially reduces the photocurrent magnitude.^48^

**Figure 3:**
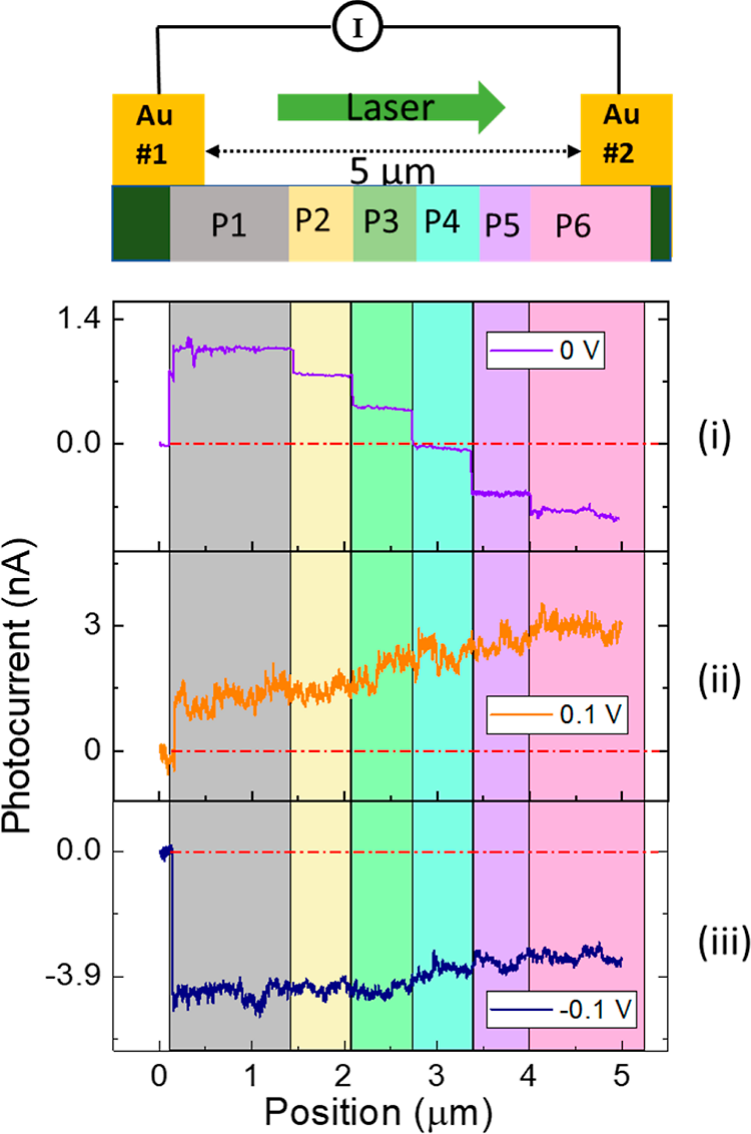
Spatially resolved photocurrents were measured in lateral Au–CrSBr–Au devices, where a linear scan of photocurrents was recorded along the direction as shown in the top panel (green arrow) while keeping the excitation laser spot at ∼500 nm in diameter that was scanning the 5 μm channel of the device. The photocurrent is given as a function of position between the two Au electrodes with an excitation laser of 633 nm at a bias voltage of (i) *V* = 0 V, (ii) *V* = 0.1 V, and (iii) *V* = −0.1 V. The top schematic is a guide to the eyes at the measured photocurrent location of the device.

A gradual change in the position of the illuminated area from the Au electrode toward the center of the device leads to reduced photocurrent as the built-in potential has the strongest value at the Au–CrSBr interface and it gradually decays (Figure 3).^48^ As expected, at the center of the device, where the distance between the excitation spot and both electrodes is the same, the magnitude of the photocurrent becomes insignificant. However, when the excitation spot came closer to the second electrode, an opposite polarity of the photocurrent was observed, and the magnitude of the photocurrent increased with a gradual approach toward the second electrode. This is because, in this state, the photocurrent is associated with the Schottky junction at the second electrode, which generates an electric field in the opposite direction. Note that the magnitude of the photocurrent varied slightly, which could be due to variations of local resistance under illumination and under dark conditions. The change in photocurrent as a function of position at 0 V bias is also shown in the Supporting Information (Figure S4). To consolidate our observation, we recorded position-sensitive measurements while applying bias (source–drain) voltage in both directions. In this case, a nearly constant photocurrent was observed for both directions of applied bias. This is because the applied bias drives the photogenerated charge carriers instead of the built-in electric field in this measurement mode. Hence, the applied bias removes the position sensitivity of the photocurrent as the applied bias has a higher magnitude than the Schottky potential. The proposed mechanism can be analytically modeled as follows.

In this device, the photon absorption and photocarrier generation are dominated by the CrSBr layer. The total number of photogenerated electrons, *n*_0_, and that of photogenerated electrons transmitted through the metal–semiconductor interface, *N*_0_, can be expressed as follows^49,50^
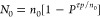
1where *P* is the probability of photogenerated electrons entering the interface, *p* is the laser power, and τ is the lifetime of the photogenerated charge carriers in the intrinsic CrSBr layer.

The electron diffusion equation at position *r* can be expressed as
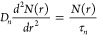
2

From these equations, the electron density at *r* and *N*(*r*) can be derived as follows
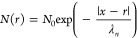
3where  is the diffusion length, , and ρ is the diffusion constant and resistivity of the semiconductor, respectively; τ_*n*_ is the electron diffusion lifetime,  is the electron density below the Fermi level (*E*_*F*0_), and *x* is the position of the laser point.

Thus, the Fermi level of the semiconductor after laser irradiation at position r can be expressed as follows
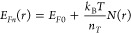
4

This transient change in charge concentration at the excitation point strongly modulates the barrier height at the interface, which drives the photocurrent. Using the above equations, an expression of the LPE at a laser irradiation position can be obtained as follows
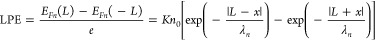
5where coefficient  and *L* and – *L* are the positions of the two electrodes. Therefore, the obtained photocurrent in the opposite electrodes has a good exhibit of symmetric values with the laser spot position, providing suitable ways of position-sensitive detection.^51,52^

### 3.3 Device Performance

**Figure 4:**
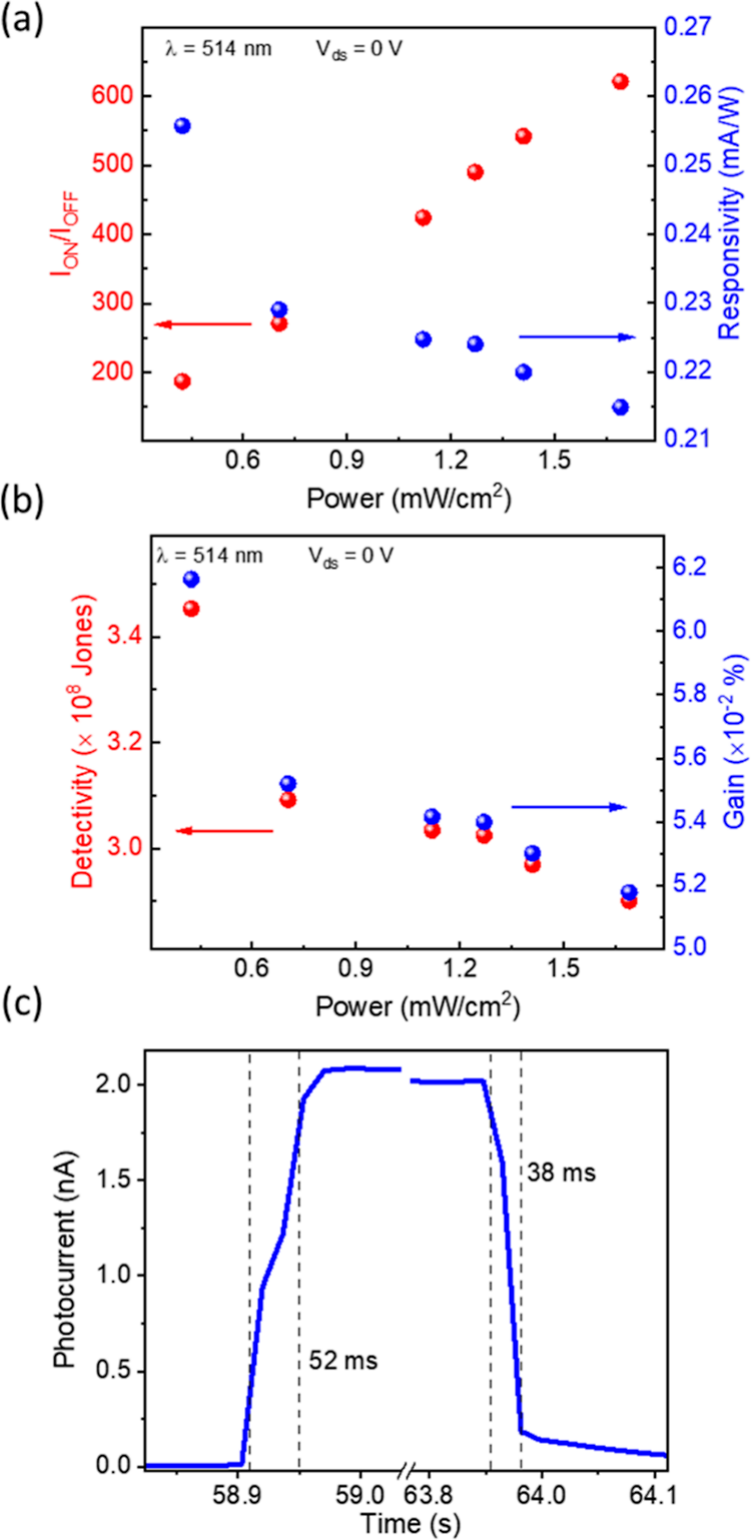
Device performance. (a) Photocurrent on/off ratio and photoresponsivity as a function of incident illumination intensity. (b) Detectivity and gain as a function of illumination intensity. (c) Photoresponse time and recovery time of the CrSBr device.

For practical applications, self-powered Schottky junction-based PDs need to meet various criteria, like an easy and cheap fabrication process, wide band photodetection, and uncomplicated integration with CMOS technologies.^14^ There are also important figures of merit to evaluate the performance of PDs, such as the photoresponsivity (*R*), detectivity (*D*), and gain (η) of the PD. We recorded the output characteristics and photocurrent of the device by systematically varying the illumination power intensity. The ratio of the extracted photocurrent to the dark current, known as the photocurrent on/off ratio, is plotted as a function of the incident power in Figure 4a. It can be seen that the photocurrent on/off ratio systematically increases with the excitation power, which is attributed to the increased density of the free e–h pairs in the CrSBr due to the band-to-band transition of the photogenerated carriers.^2,53^ Note that the measured on/off ratio is limited by the sensitivity of our current source meter, which allows the dark current to be measured only down to 1 nA. Broadband photoresponse of the devices has been studied utilizing 488, 514, 568, and 633 nm excitation sources of an Ar–Kr laser. The obtained photoresponse under a constant power density has been provided in Figure S5. The photoresponsivity of the detector is defined as the photocurrent generated per unit incident power on the effective area of the PD, which can be expressed as , where *I*_ph_ is the net photocurrent and *P* is the incident power on the device.^2,53^Figure 4a presents the calculated photoresponsivity as a function of the incident power density at zero bias voltage. The highest value of the photoresponsivity found was ∼0.26 mA/W for 0.42 mW/cm^2^ at zero bias voltage. The value of *R* shows a negative dependence on the incident power, which is consistent with the previously obtained results.^7,54,55^ With increasing illumination intensity, the number of photocarriers (e–h pairs) increases, which induces more recombination of the photogenerated excitons due to high exciton binding energy.^56,57^

The detectivity describes the smallest detectable signal. Assuming that shot noise is the major factor contributing to the dark current, the detectivity can be expressed as , where *R* is the photoresponsivity, *A* is the area of the incident light on the PD, *e* is the elementary charge, and *I*_d_ is the dark current. The variation of the detectivity of the PD with the incident power density is shown in Figure 4b. The value of *D* decreases with increasing incident power and the maximum value of *D* obtained was ∼3.4 × 10^8^ Jones. The change of *D* with laser power can be explained by the linear dependence of *D* on *R* and a constant dark current. Note that the laser heating of the device channel has been assumed to be negligible.^2,58^

The photocurrent gain is a dimensionless figure of merit of the device given by the number of photoexcited carriers generated per unit photon. Assuming that all incident photons are absorbed in the device active area, η can be expressed as , where *h* is the Planck constant, *c* is the speed of light in free space, and λ is the wavelength of the incident photon beam. The variation of the gain as a function of the incident power is shown in Figure 4b. The maximum value of η was ∼6.2 × 10^–2^ % at the pump fluence of 0.42 mW/cm^2^ in self-powered conditions, thus demonstrating the relatively high efficiency of the device for light detection.^46^ The transient photoresponse and recovery time of the devices have been found to be nearly 52 and 38 ms, respectively, which is faster than typical monolayer semiconductor transition metal dichalcogenide-based devices, where a prolonged photoresponse in planar configuration is observed.^56,59^

### 3.4 Polarization-Resolved Photodetection at the Ambient Conditions

**Figure 5:**
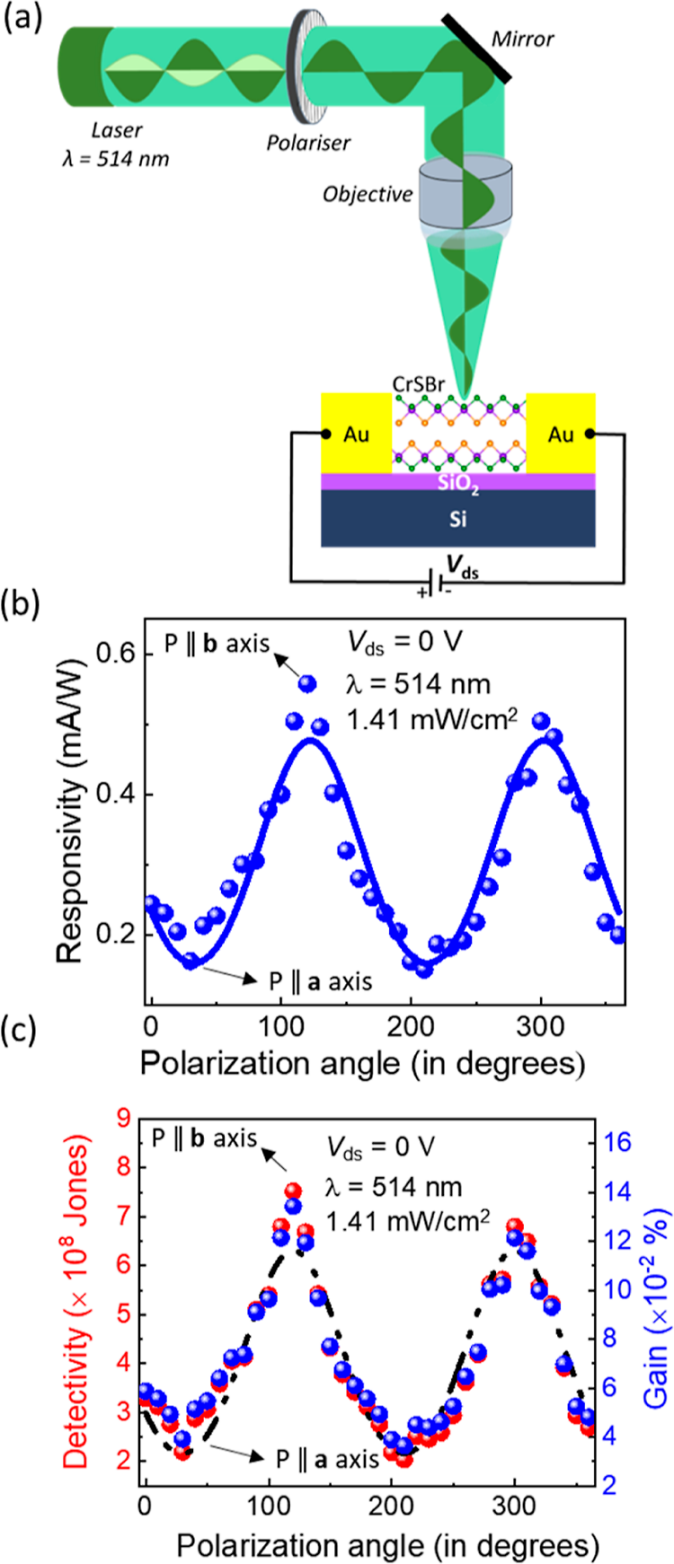
Polarization-resolved self-powered device performance. (a) Schematic illustration of the measurement setup where the excitation laser was linearly polarized by a half-wave plate before the laser entered the confocal microscope. We monitored the polarization at the output of the objective of the microscope. Additionally, we carefully recorded the excitation power to keep it at a constant value to obtain the optical polarization-induced variation. (b) Photoresponsivity as a function of different polarization angles. The blue curves indicate the results of fitting to the (*a*^2^ sin^2^θ + *c*^2^ cos^2^θ)^2^ function, where θ is the polarization angle. (c) Detectivity and gain as a function of polarization angle.

CrSBr is suitable for high-sensitivity polarization detection due to its low-symmetry crystal structure, which is similar to that of black phosphorus,^60^ CrPS_4_,^61^ and GeSe_2_.^62^ Thus, we performed further polarization-dependent photocurrent measurements on the same devices under the excitation of a linearly polarized 514 nm photon beam. A schematic illustration of the experimental setup is given in Figure 5a. The details of the experimental setup are discussed in the Methods Section. The photocurrent was measured under the excitation of the polarized laser beam while varying the angle of the incident from 0 to 360° with respect to the crystalline axes of the single crystalline CrSBr. The step interval was 10° and no external bias was applied. Note that the excitation laser illuminated the area next to one of the Au electrodes throughout the whole measurement. We further evaluated the corresponding angle-dependent photoresponsivity, gain, and detectivity, as shown in Figure 5b,c, respectively, which exhibit quasi-sinusoidal behavior with respect to the polarization angle of the incident photon beam. We further calculated the photocurrent anisotropic ratio *r* = (*I*_ph-max_ – *I*_ph-min_)/(*I*_ph-max_ + *I*_ph-min_) and the dichroic ratio *d* = *I*_ph-max_/*I*_ph-min_, which were found to be 0.65 and 3.43, respectively, for an incident power density of 1.4 mW/cm^2^, indicating very high sensitivity to the polarization of the incident photons even at the self-powered conditions.

### 3.5 Polarization-Resolved PL at the Ambient Conditions

**Figure 6:**
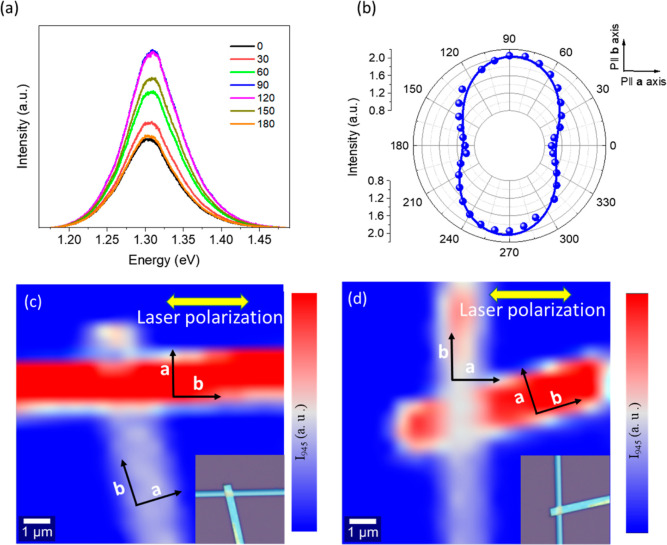
Polarization-resolved PL of a few-layer CrSBr under the illumination of 514 nmfrom 0 to 180 degrees. (a) Linearly polarized PL spectra of an 82 nm CrSBr flake at different polarization angles of the excitation source. (b) Polar plot of the angle-resolved PL peak intensity at 1.31 eV. The blue curve indicates the results of fitting to the (*a*^2^ sin^2^θ + *c*^2^ cos^2^θ)^2^ function, where θ is the polarization angle. (c,d) Spatial distribution of integrated PL intensity of a nearly perpendicular CrSBr heterostructure on the SiO_2_/Si substrate, where the excitation laser polarization was kept fixed and the sample was rotated by 90°. The **b**-axis of the flake parallel to the laser polarization shows high luminescence for a particular angle (0° for the top flake and 90° for the bottom flake). The inset is an optical image of the CrSBr films situated in a nearly perpendicular orientation.

We also performed the polarization-dependent PL measurement under ambient conditions. Figure 6a shows the PL spectra recorded for the excitation of the 514 nm laser beam at different polarization angles with respect to the crystalline axes of the CrSBr. The origin of the PL peak centered at 950 nm (∼1.31 eV) can be traced to 1s excitonic recombination, as suggested previously.^63^ In Figure 6b, we showcase a polar plot of the PL intensity of the 1s exciton peak, which clearly exhibits a dumbbell-shaped anisotropic nature with a 180° variation period and intensity maxima at 90 and 270° (along the **b**-direction). It can be seen that the optical band-to-band transitions are allowed only along the **b**-direction. To understand this peculiar directional dependence of the PL, one has to consider the electronic band structure of CrSBr.^32,33,63^ It is known from the theoretical calculations that the large band gap possesses highly pronounced anisotropy in the Γ–*Y* direction (along the **b**-direction of the crystallographic axis), which vanishes completely along the Γ–*X* direction (along the **a**-direction of the crystallographic axis).^14,15,36^ This implies that interband transitions are forbidden along the **a**-axis from the conduction band maxima and valence band minima at Γ.^32,33,63^ We further performed polarization-dependent spatially resolved PL measurements with a 532 nm excitation. For this experiment, we used two nearly perpendicular CrSBr flakes exfoliated on the SiO_2_/Si substrate, as shown in the insets of Figure 6c,d. In Figure 6c, the incident laser polarization is aligned with the flake orientation at the top along the **b**-axis and hence we observe strong luminescence, while it is negligible for the flake at the bottom in a perpendicular direction. For the experiment shown in Figure 6d, we kept the polarization of the laser fixed and rotated the sample stage by 90° (see the insets of Figure 6c,d). For this scenario, we observed that the **b**-axis of the flake at the bottom was now aligned to the polarization of the incident laser, and it showed strong luminescence while the top flake did not.

### 3.6 Polarization-Resolved Raman Spectroscopy

Raman spectroscopy is a powerful tool to characterize anisotropic materials. The scattered light intensity (*Ĩ*) is dependent on the polarization of the incident light (*e*_i_) and the scattered light (*e*_s_) via the Raman tensor (Γ) for the corresponding Raman modes. According to the Placzek model, the Raman scattering intensity is given by *Î* ∝ |*e*_i_Γ·*e*_s_|^2^, where *e*_i_ and *e*_s_ are the polarization unit vectors for the incident and scattered light and Γ is the Raman tensor for the Raman active modes.^60,64^ Hence, it is possible to extract information about the crystallographic anisotropy for any material by considering the polarization-dependent Raman response.

**Figure 7:**
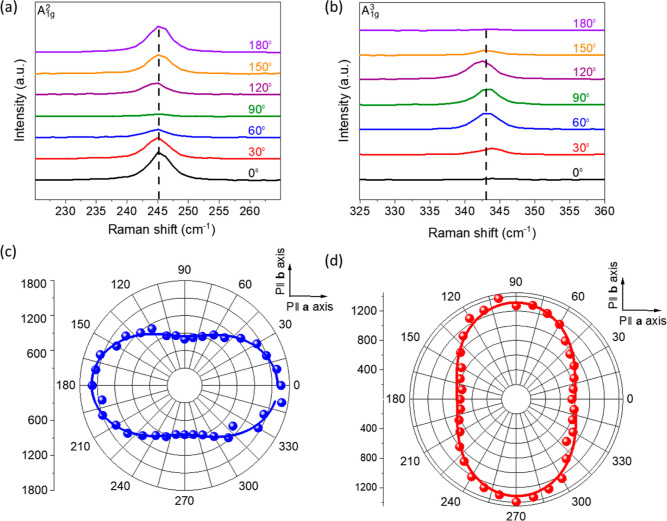
Anisotropic phonon scattering. (a) Excitation angle-dependent linearly polarized Raman signals of the A_1g_^2^ mode around 245 cm^–1^ and (b) A_1g_^3^ mode around 343 cm^–1^ of a multilayer CrSBr flake. (c) Polar plot of the integrated Raman intensity of the A_1g_^2^ mode and (d) A_1g_^3^ mode recorded under different angles of the incident laser polarization with the crystal axes. The blue and red curves indicate the results of fitting to the  function, where θ is the polarization angle.

Figure 7 shows the polarization-dependent Raman spectra of multilayer CrSBr flakes. Figure 7a,b shows stack plots of the polarization-dependent Raman spectra covering both **a** and **b** directions for A_1g_^2^ and A_1g_^3^ at the 245 and 343 cm^–1^ modes, respectively. The corresponding polar plots of the two principal Raman modes, A_1g_^2^ and A_1g_^3^, are shown in Figure 7c,d. The measurement was carried out by coaligning the linearly polarized excitation light with the detector polarization. We observe that the modes A_1g_^1^ (not shown here) and A_1g_^3^ for our multilayer CrSBr crystal show anisotropic vibrational response (dumbbell-shaped) with a 180° variation period and intensity maxima at 90° and 270° along crystallographic orientation **b** (direction **b**). However, the A_1g_^2^ mode is an exception, as it is observed to be rotated by 90° as the scattered intensity shows maxima corresponding to 0 and 180° along crystallographic orientation **a** (direction **a**) with a 180° variation period. The anisotropic polarization response in this case for the A_1g_^2^ mode along the **a**-axis can be attributed to the quasi-1D-like electron–phonon interaction based on the electronic structure of CrSBr, which is strongly influenced by coupling of the high-density states of the electronic system along the symmetrical Γ–*X* direction. This anisotropic directional dependence of the different Raman modes is consistent with recent observations of the single crystal of CrSBr having an orthorhombic structure with D_2h_ symmetry.^63^ The observable out-of-plane A_1g_ modes can be well fitted with the equations of the Raman scattering intensity obeying the relation (*s*^2^ sin^2^θ + *c*^2^ cos^2^θ^2^) for the parallel configuration. This corresponds to the experimental setup, in which the polarization direction of the scattered light is normal to the plane without the presence of the B_g_ Raman modes.^25^ Hence, the obtained PL and Raman spectra corroborate the anisotropic responses of the A_1g_^2^ and A_1g_^3^ Raman modes of the crystal. Thus, we confirm that the polarization-dependent behavior is an intrinsic property of the CrSBr.

**Figure 8:**
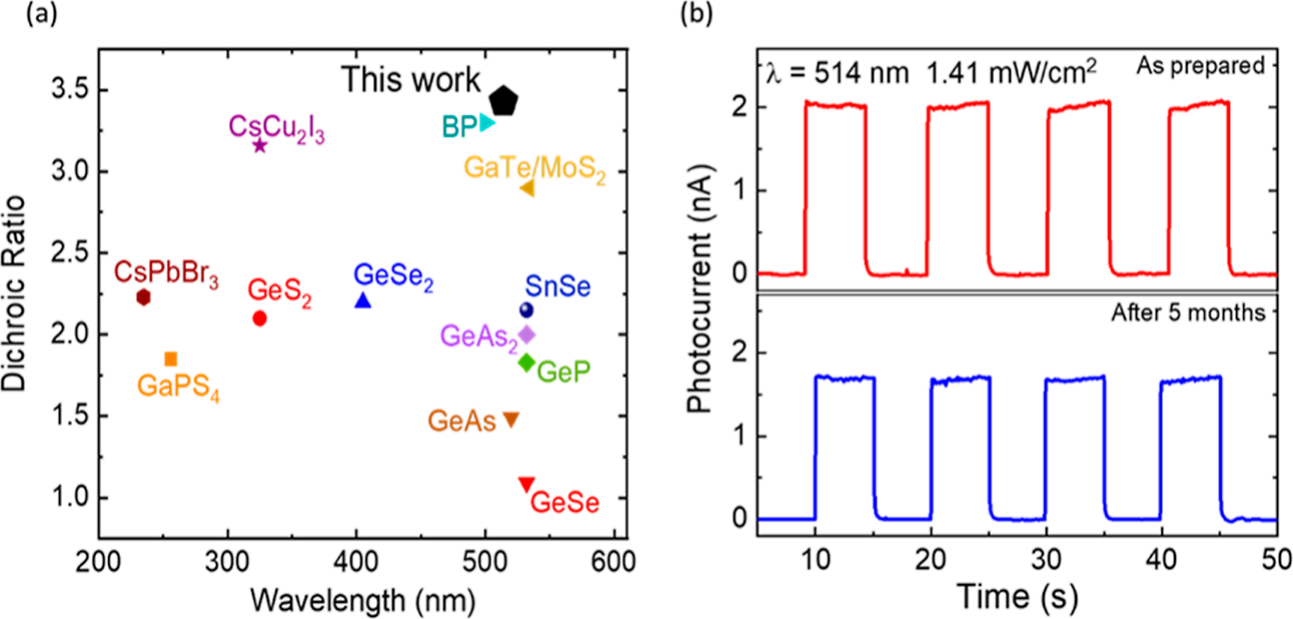
Comparison of the dichroic ratios achieved by the polarized photocurrent measurement and the stability of the device. (a) Photocurrent dichroic ratio of the CrSBr with respect to the reported values of the devices based on other 2D materials (BP: black phosphorus). (b) Transient photoresponse of the same device measured after 5 months.

In addition, the obtained *r* and *d* values of our PD are larger than those for previously reported devices based on other 2D materials.^62,65−72^ In Figure 8a, we have presented a comparison of the reported dichroic ratio of the PD devices based on few-layer 2D materials, for which our CrSBr device attains a value of 3.4, one of the highest reported so far. The measured devices exhibit a highly stable and reproducible performance. To test the long-term stability of the device, we have also recorded photocurrent data under ambient conditions after a period of ∼5 months (Figure 8b). We did not observe any significant change in the device performance during this period. These results thus demonstrate the great potential of 2D CrSBr for application in self-powered linear dichromatic optoelectronic devices.

## 4 Conclusions

We studied CrSBr crystals as a promising air-stable, self-powered, polarization-sensitive PD. The detector is based on a multilayer CrSBr/Au Schottky junction prepared by mechanical exfoliation and a dry transfer method. The photocurrent was measured in the absence of external bias under varying incident light power, yielding a photoresponsivity value of ∼0.26 mA/W at an incident light power of 0.42 mW/cm^2^. The photodetectivity of the device was about 3.4 × 10^8^ Jones and the gain was η = 6.2 × 10^–2^ % at zero bias voltage. The photoresponse of the devices was found to be position-dependent due to the different electric fields across the junction. The anisotropic ratio of the device was estimated to be ∼0.65 and the dichroic ratio was estimated to be around 3.4. The high degree of anisotropy in photodetection was caused by the puckered crystal structure of CrSBr, which caused a break of the sublattice symmetry. These results clearly demonstrate that CrSBr is a promising anisotropic 2D material for the development of high-quality polarization-dependent PDs for imaging and optical communication applications.

## Supporting Information Available

Layered-dependent Raman and PL spectra of CrSBr, *I*–*V* characteristic of CrSBr with and without illumination, bias-dependent photocurrent, position-sensitive photocurrent at 0 V, and wavelength-dependent photocurrent (PDF)
