# Supplementary material for: Polarization-Resolved Position-Sensitive Self-Powered Binary Photodetection in Multilayer Janus CrSBr
Source: ACS Appl Mater Interfaces. 2023 Dec 26;16(1):1033–43. doi: 10.1021/acsami.3c13552 (PMC10788859; doi:10.1021/acsami.3c13552)
Supplement: Supplementary file 1 — am3c13552_si_001.pdf [file am3c13552_si_001.pdf]

Supporting Information

Polarization-Resolved Position-Sensitive Self-Powered Binary Photodetection in Multilayer Janus CrSBr

Jaganandha Panda[1], *Satyam Sahu*[1,2], *Golam Haider*[1*], *Mukesh Kumar Thakur*[1], *Kseniia Mosina*[3], *Matěj Velický*[1], *Jana Vejpravova*[4], *Zdeněk Sofer*[3], *and Martin Kalbáč*[1*]

1. J. Heyrovský Institute of Physical Chemistry, Dolejskova 3, 182 23 Prague 8, Czech Republic

2. Department of Biophysics, Chemical and Macromolecular Physics, Faculty of Mathematics and Physics, Charles University, Ke Karlovu 3, 121 16 Prague 2, Czech Republic

3. Department of Inorganic Chemistry, University of Chemistry and Technology Prague, Technicka 5, 166 28 Prague 6, Czech Republic

4. Department of Condensed Matter Physics, Faculty of Mathematics and Physics, Charles University, Ke Karlovu 5, 121 16 Prague 2, Czech Republic

*Email: haider.golam@jh-inst.cas.cz and martin.kalbac@jh-inst.cas.cz

Layer-dependent photoluminescence and Raman spectra of CrSBr

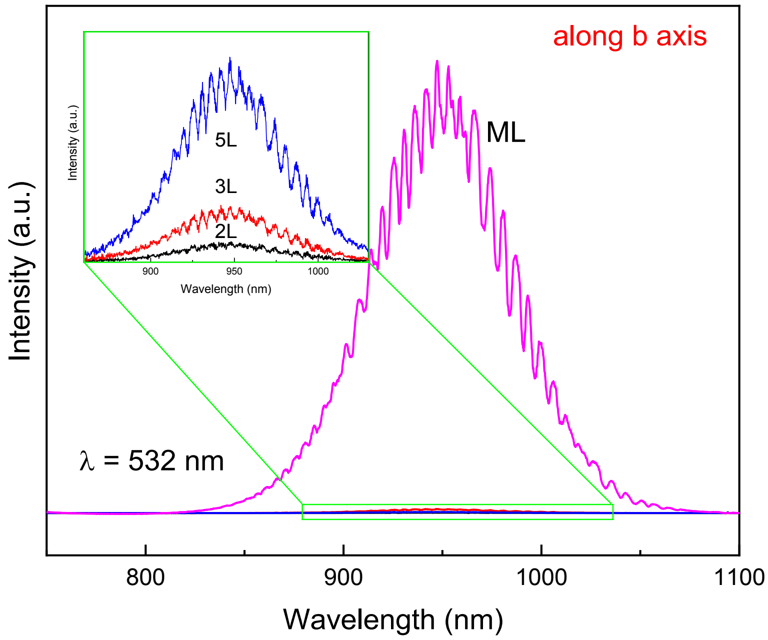

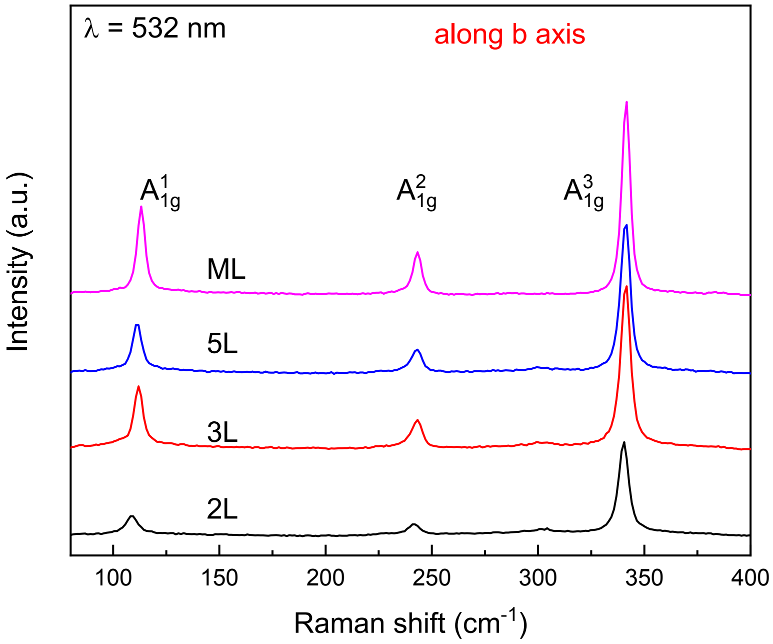

Figure S1. Layer dependent photoluminescence and Raman spectra of CrSBr

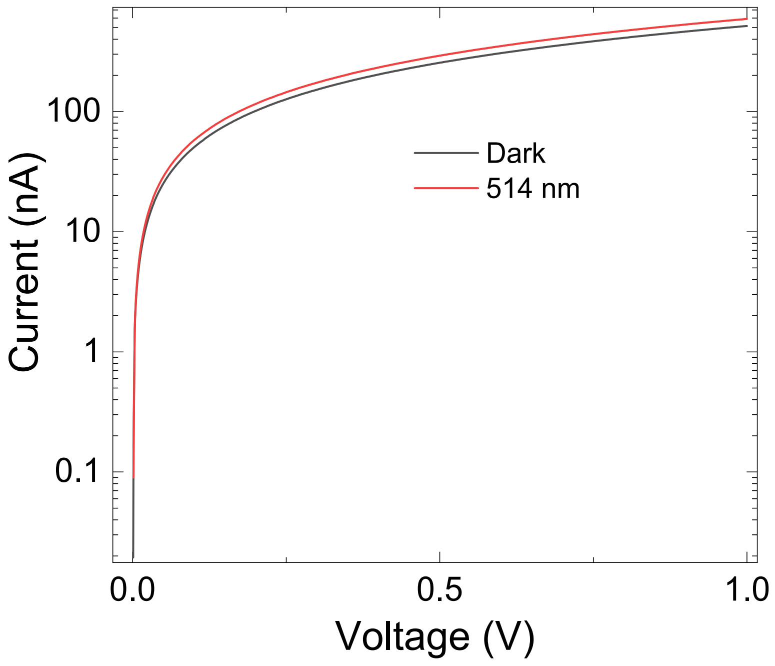

Figure S2: I-V characteristics of the self-biased device under dark and illumination of 514 nm excitation of intensity 0.70 mW/cm^2.

External bias dependent photocurrent

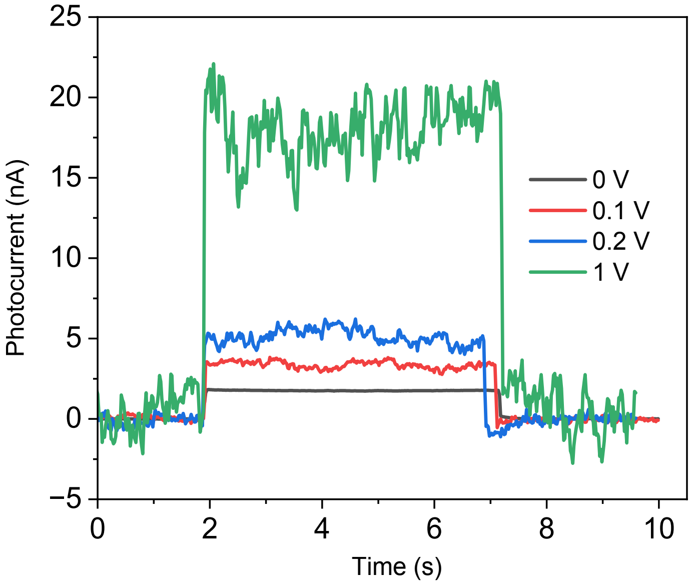

Figure S3. External bias dependent photocurrent of CrSBr under the excitation of excitation power of 1.41 mW/cm^2 for 514 nm wavelength.

Position-sensitive photocurrent in CrSBr

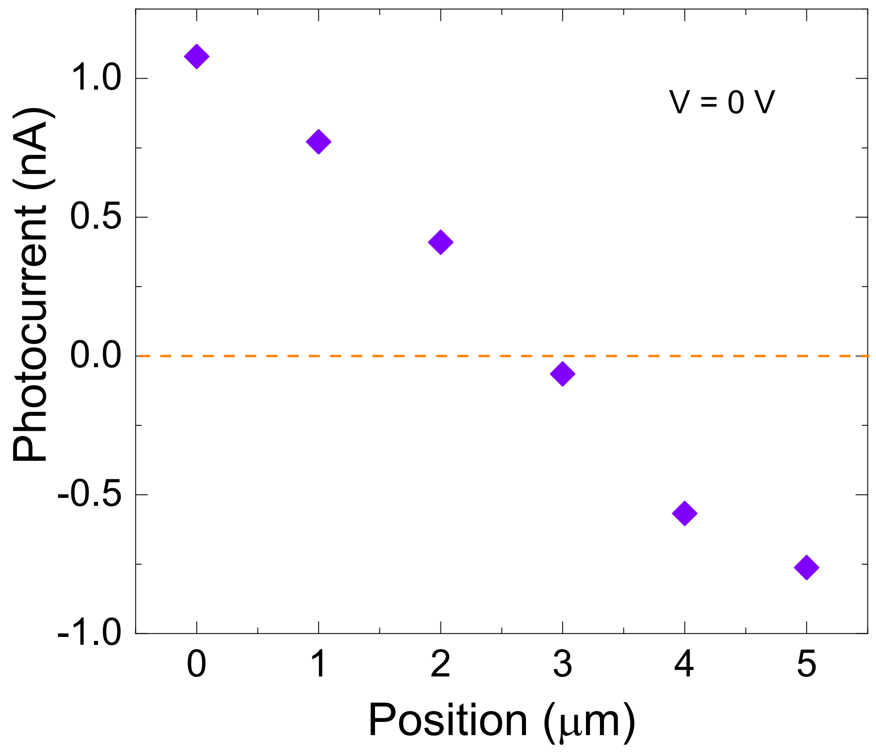

Figure S4. Variation in photocurrent as a function of position between two Au electrodes under 633 nm excitation at 0 V bias.

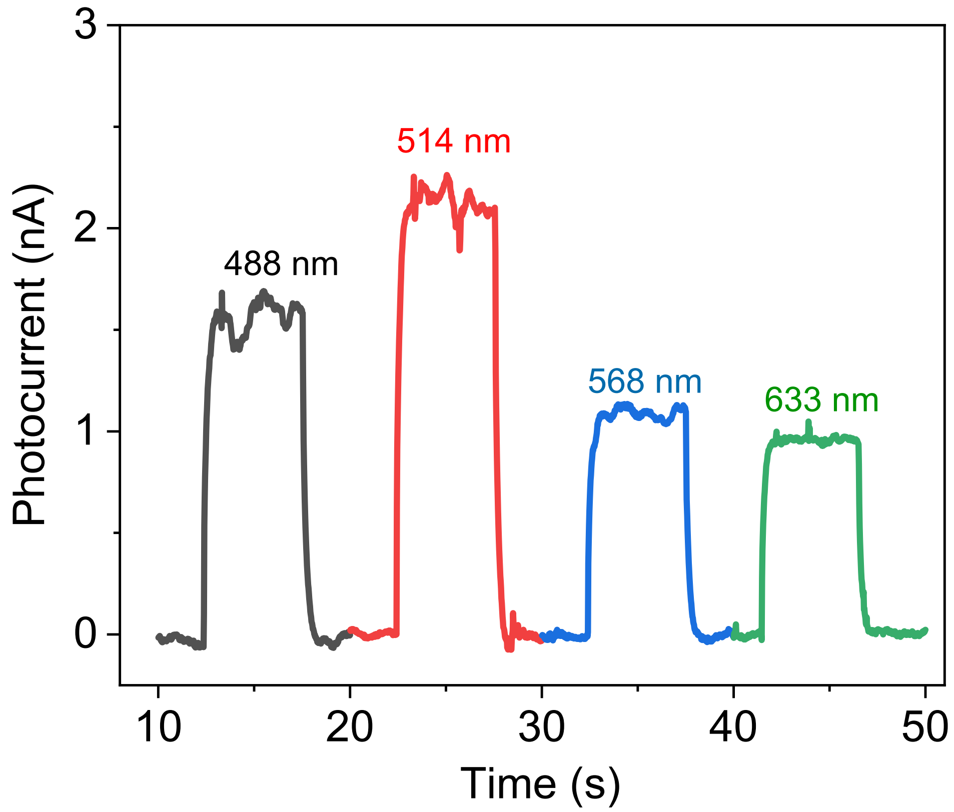

Figure S5: Transient photocurrent of the device under the illumination of different excitations power of 1.69 mW/cm^2 ranging from 488 nm to 633 nm, resembling broadband detection.
